# How does qualitative data collection modality affect disclosure of sensitive information and participant experience? Findings from a quasi-experimental study

**DOI:** 10.1007/s11135-021-01217-4

**Published:** 2021-09-02

**Authors:** Emily Namey, Greg Guest, Amy O’Regan, Christine L. Godwin, Jamilah Taylor, Andres Martinez

**Affiliations:** 1 FHI 360, Behavioral, Epidemiological, and Clinical Sciences, Durham, NC USA; 2 Pacific Institute for Research and Evaluation (PIRE), Chapel Hill, NC USA; 3 Department of Population Health Sciences, Duke University School of Medicine, Durham, NC USA

**Keywords:** Qualitative data collection, Online qualitative research, Sensitive information, Research participant experience

## Abstract

Focus groups (FGs) and individual interviews (IDIs) can be conducted in-person or in several different online contexts. We conducted a quasi-experimental study and assessed sharing of sensitive or dissenting information and participant comfort in FGs and IDIs across four modalities: (1) in-person, (2) online video-based, (3) online chat-based (synchronous), and (4) online email/message board-based (asynchronous). Participants were systematically assigned to one of the four modalities and randomized to one of 24 FGs or 48 IDIs (*N* = 171). The study topic was medical risk during pregnancy. All participants also completed a survey on their perceptions of the data collection process. We found no significant difference in the frequency of disclosure of sensitive information by modality. Text-based FGs (chat and message board) were more likely to contain dissenting opinions than visually-based FGs (in-person and video). Participants also reported feeling less rapport and personal comfort in sharing information in the FG video modality than other modalities. These findings provide initial data that can guide researchers in choosing among data collection modalities to maximize participant engagement and comfort.

## Introduction

Prior to the COVID-19 pandemic, online focus groups (FGs) and individual interviews (IDIs) were being used with increasing frequency across multiple disciplines (Braun et al. 2017; Salmons 2016), a trend that the pandemic has accelerated and mainstreamed, at least for the near-term (Saarijärvi and Bratt 2021; Rahman et al. 2021). A key advantage of online data collection has always been that a researcher can collect data remotely and from individuals across multiple locations (Comley and Beaumont 2011). Eliminating the need to travel or meet in person also extends data collection to marginalized populations (Reisner et al. 2018) or to those for whom travel might be difficult (Tates et al. 2009). Online modalities have been shown to work well with populations facing unique health challenges such as traumatic brain injury (Egan et al. 2006), autism (Benford and Standen 2011), multiple sclerosis (Synnot et al. 2014), and chronic conditions (Nicholas et al. 2010).

Online qualitative data collection modalities can be categorized along two dimensions. One dimension refers to the nature of communication medium—text or video. In a text-based modality, questions and responses are typed via computer. Video-based modalities use online video (with audio) technology and questions/responses are spoken. The other dimension pertains to temporality—synchronous or asynchronous. Synchronous methods are conducted in real-time, typically through text- or video-conferencing (Fox et al. 2007). Conversely, asynchronous methods occur in a back-and-forth manner over a period of time, and are typically conducted through discussion boards or listservs (Rezabek 2000). Synchronous methods tend to be relatively fast-paced with rapid communication flow, whereas asynchronous methods allow participants more time to consider and respond to questions. The latter are purported to generate richer and deeper data (Fox et al. 2007; Oringderff 2004).

As online qualitative data collection becomes more common, it is important to understand what the trade-offs of moving from in-person to remote data collection might be. Recent empirical studies designed to compare features of qualitative datasets generated in-person and online have found some differences in the amount of data provided by participants, but very little difference in the thematic content of responses (Krouwel et al. 2019; Namey et al. 2020). In terms of the effects of data collection modality on specific aspects of the data, the limited empirical research on disclosure of sensitive topics, expression of dissenting opinions, and participant experiences of different modes for focus group research are summarized in Table 1. The findings of these studies, along with similar work on comparisons of quantitative surveys, suggests that individuals are more likely to express socially unsanctioned or highly personal (“sensitive”) opinions or behavior in online settings (Campbell et al. 2001; Walston and Lissitz 2000; Turner et al. 1998; Spector 2004), a phenomenon described as the “online disinhibition effect” (Suler 2004). Researchers have found similar results of greater online openness when comparing focus group participants’ willingness to express dissenting or contrary opinions (Massey and Clapper 1995; Underhill and Olmsted 2003; Reid and Reid 2005; Graffigna and Bosio 2006). Of the studies that compared participant satisfaction between or among modes of data collection, there was no consensus finding on how participants preferred to connect (Reid and Reid 2005; Nicholas et al. 2010; Massey and Clapper 1995; Underhill and Olmsted 2003; Walston and Lissitz 2000).

**Table 1:** Summary of literature comparing in-person to online focus groups on sensitive disclosures, dissenting opinions, and participant experience

Characteristic	Modality*			
	In-person	Online synch text	Online asynch text	No differences
More discussion/disclosure of sensitive topics		Massey and Clapper (1995) Walston and Lissitz (2000) Campbell et al. (2001) Graffigna and Bosio (2006) Woodyatt et al. (2016)**	Nicholas et al. (2010) Graffigna and Bosio (2006)	Reid and Reid (2005)
More dissenting opinions/ disagreement			Massey and Clapper (1995) Underhill and Olmsted (2003) Graffigna and Bosio (2006) Reid and Reid (2005)	
Greater participant satisfaction	Reid and Reid (2005) Nicholas et al. (2010)	Massey and Clapper (1995)		Underhill and Olmsted (2003) Walston and Lissitz (2000)

*Online video is omitted from this table as no prior peer-reviewed studies comparing these characteristics in focus groups were found. Archibald et al. (2019) found equal or greater participant satisfaction for Zoom-based individual interviews compared to in-person, but did not conduct a head-to-head comparison

Nearly all of these studies comparing online and face-to-face modes of data collection are limited to focus groups, however, and few employed an experimental design. Many did not control for instrument or interviewer variation, had very small sample sizes, featured only one type of online data collection, and/or lacked systematic and transparent analytic procedures. And, despite many articles reflecting on advantages and disadvantages and lessons learned about online qualitative research before and since the pandemic (Weller 2015; 2017; Janghorban et al. 2014; Corti and Fielding 2016; Vindrola-Padros et al. 2020; Lobe et al. 2020), few actually compare data generated or participant experiences with the different modalities, as Weller (2015) notes.

In early 2016 we designed a quasi-experimental design to address these gaps in the literature. Participants were randomized to either an IDI or FG arm and systematically assigned to one of four modes of data collection: (1) face-to-face, (2) video-based, (3) synchronous text-based, or (4) asynchronous text-based. The analyses we present in this article focus specifically on comparing the amount of sensitive data, dissenting opinions in focus groups, and participant satisfaction with face-to-face and remote modes of data collection. Findings on thematic content generated across modalities and comparative costs are reported elsewhere (Namey et al. 2020).

### Study context

The research presented here was funded by the Patient Centered Outcomes Research Institute (PCORI), which funds methodological research in the interest of generating better data to inform the health needs of patient populations. Accordingly, though the primary aims of our study were methodological, we chose a topic of public health relevance at the time to guide our sample selection and inform our study instrument: we examined women’s perceptions of medical risk and research during pregnancy, with a focus on the Zika virus.

## Methods

### Study design

We conducted a quasi-experimental, cross-sectional qualitative study in which participants were randomly assigned to focus groups or individual interviews and systematically stratified into one of four data collection modalities, per Table 2.

**Table 2:** Eight Study Arms by Mode of Data Collection, Timing, and Method

Data Collection Modality	Communication Type	Timing	Data Collection Method	
			Focus Groups	Individual Interviews
Face-to-Face (in-person)	Verbal/visual	Synchronous	*n* = 6 groups	*n* = 12
Online: Video-based	Verbal/visual	Synchronous	*n* = 6 groups	*n* = 12
Online: Chat-based (text)	Text/typing	Synchronous	*n* = 6 groups	*n* = 12
Online: Email/message board	Text/typing	Asynchronous	*n* = 6 groups	*n* = 12
Total data collection events			24 groups	48 interviews

The number of data collection events for each of the cells in Table 2 was based on the minimum number of data collection events we estimated would be required to reach 80–90% data saturation per method of data collection, based on previous empirical research (Guest et al. 2006, 2017, Namey et al. 2016). We opted to include six focus groups per modality (more than the 2–3 suggested as sufficient to reach saturation (Namey et al. 2016; Guest et al. 2017a) to provide more data points and variability to ensure we could meaningfully compare data collection modalities.

**Fig. 1:**
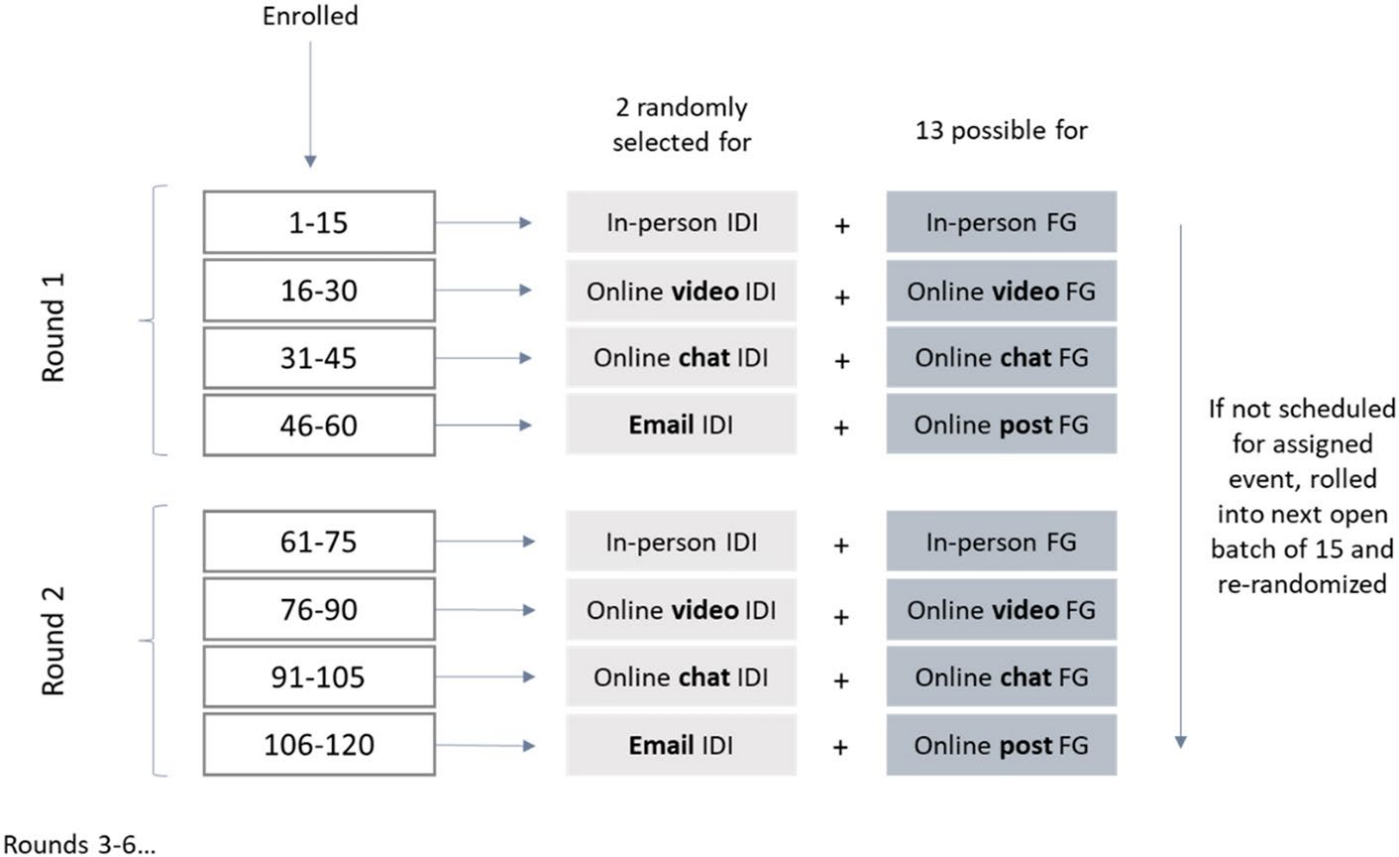
Systematic assignment of participants to modality and method (reprinted from Namey et al. 2020)

To avoid temporal bias, data collection was conducted in a successive manner, completing one “round” of data collection for each modality before repeating the process. There were six rounds of data collection, each included two IDIs and one FG per modality (Fig. 1).

### Sampling, recruitment, and randomization

We recruited women over age 18 who had been pregnant between 2013 and 2016 and who were hoping to become pregnant again in the next three years at the time of enrollment. Additionally, given the online nature of three of the data collection modalities, women enrolled had to have access to the internet and self-reported typing skills, and had to agree to randomization to either an IDI or FG and assignment to either online or in-person data collection.

We recruited participants through a combination of online social media, local community events, magazine advertisements, radio announcements, and flyers posted near establishments that serve pregnant and postpartum women. Because randomization of women to mode of data collection was logistically challenging given the need for a certain number of participants per FG and the temporal design of the study, we instead systematically assigned women to a modality in a way that would limit the introduction of bias. As women consented to participate and were enrolled in the study, the first 15 women were assigned to a face-to-face modality. Once the scheduling list for face-to-face events was full, the next 15 women to enroll were assigned to the video modality, and so on through each of the four modalities for Round 1 (see Fig. 1). Each group of 15 women were then randomized in a weighted manner according to a computer-generated sequence: 2 to take part in IDIs and 13 to be included in the scheduling pool for a FG. Once all Round 1 scheduling lists were full, the process was repeated for Round 2. Any women who were enrolled but not scheduled were rolled over into the next scheduling list and re-randomized. Women were blinded to their assignments until they were scheduled.

### Data collection

We collected data from September 2016 to October 2017. Given the comparative nature of the study, we attempted to control for potential confounders by minimizing differences in the data collection processes. The data collector (an experienced female interviewer) and the data collection guide were identical across all modalities. Procedures, aside from technical connection requirements, were also kept consistent within each modality in line with their respective best practices (Wilkerson et al. 2014). Questions on the instrument were open-ended and asked in the same order, to enhance comparability (Guest et al. 2013). As with any qualitative inquiry, the data collector asked inductive follow-up questions based on participant responses, but did not introduce ideas from any previous data collection event. In addition to questions about women’s perceptions of safe behaviors during pregnancy, clinical research, and Zika, and to ensure some discussion of a potentially sensitive topic, we also included a series of questions on abortion as related to the larger topic areas. Prior to implementation, the data collection instrument was pilot-tested among consented participants, to enhance clarity and validity of questions, as well as to trouble-shoot potential technological issues.

At the end of each data collection event participants completed a brief anonymous questionnaire containing several structured questions on their perceptions of the activity. Questions pertained to women’s opinions about how comfortable and safe they felt sharing information through their particular data collection modality. In the FG contexts, the structured questions were completed individually and independently from the group. All face-to-face and video-conferencing data collection activities were digitally audio-recorded. Audio recordings of FGs and IDIs from the in-person and online video modalities were transcribed following a standardized protocol (Guest et al. 2013; McLellan et al. 2003). Transcripts for the online text-based activities were automatically created as part of the data collection process. Details on data collection procedures for each modality are included in the endnotes.^1^

### Data analysis

We employed both deductive and inductive thematic analyses, facilitated by NVivo 11 (QSR 2015). We first created a priori codes for “sensitive/personal” disclosures, to allow comparison across data collection modalities in terms of the frequency of these types of themes. The definition and process for identifying these disclosures were derived from a previous study with a similar aim (Guest et al. 2017b). We also created an a priori “dissenting opinion” code for focus group data, to capture instances in which a participant expressed an opinion opposite to an opinion expressed by another participant earlier in the discussion. Our definitions for these two codes were:

**Table:** 

Sensitive/Personal	Information about one’s own experience that is highly personal, taboo, illegal, or socially stigmatized in nature, which we would reasonably expect people to be reluctant to disclose to a stranger(s)
Dissenting Opinion	In a focus group, participant expresses an opinion that counters or contradicts an opinion(s) that has been previously offered by another participant in the discussion

As data were reviewed, these a priori codes were applied to any sections of text that met their definition.

#### Sensitive/personal themes and dissenting opinions

To compare the frequency of sensitive disclosures in online and face-to-face modalities, we inductively reanalyzed the data coded as “sensitive” to identify the types of unsolicited sensitive/personal themes disclosed. We summed and compared the number of unique transcripts in which sensitive themes appeared and were coded.

To assess whether participants were more likely to offer a dissenting opinion in an online versus face-to-face FG, we analyzed data from one FG question that asked about the effect of Zika on women’s personal views on abortion. This topic was selected with the assumption that most women have a view on abortion that they have thought about prior to their focus group participation and that it intersected with the rest of the discussion on pregnancy and risk perception in the context of Zika. Additionally, all women were asked to indicate their position on abortion as part of a self-administered closed-ended demographic information survey that preceded the focus groups and interviews, so were primed to have thought about their opinion.

Focus group responses to the abortion question were categorized by: (a) whether any member of the group dissented from a previously stated opinion of others in the group (dissension), and (b) the number/percent of participants choosing to abstain from answering this question (abstention). These data were then compared across modality of focus group.

To enhance analytic reliability, all transcripts were independently coded by two data analysts for all steps described above. Inter-coder agreement checks were carried out on each transcript. After each of these checks, analysts discussed any coding discrepancies. All discrepancies were resolved, and a master transcript was coded to reflect the agreed-upon changes.

#### Participant experiences

We assessed participants’ experiences of data collection both quantitatively and qualitatively. Participants’ responses to a series of questions with Likert-scale response options were tabulated by modality. Comments provided in an open text box associated with each question were reviewed and summarized to augment interpretation of the quantitative data.

#### Statistical analyses

We tested all outcome measures for differences by modality, separately for FGs and IDIs. For some analyses, we also considered audio-visual methods (face-to-face and online video) compared to text-based online methods, where the visual connection of the method may have been more important than whether it was on- or offline. For the dichotomous outcomes—sensitive disclosure and dissenting minority opinion—we used a Chi-square test; and for the responses on a Likert scale we used a Kruskal–Wallis test. All data preparation and quantitative analyses were conducted using SAS Enterprise Guide 7.1.

### Research ethics

The study was reviewed and approved by FHI 360’s Protection of Human Subjects Committee, and verbal informed consent was provided by all participants before initiation of data collection.

## Results

### Sensitive themes

The thematically recoded data from the a priori “sensitive” code reflected the nature of sensitive disclosures. These included mention of drinking some amount of alcohol while pregnant; taking medication for anxiety, depression, or other mental health condition; smoking cigarettes while pregnant; being exposed to marijuana smoke while pregnant; and having had a previous abortion.^2^ We did not directly solicit information on these topics. Frequencies of all disclosures are described at the individual level for IDIs and at the group level for FGs (since there is not response-independence in a group, we count only the first disclosure); some data collection events had more than one sensitive disclosure.

Across the individual interview data, three of these types of sensitive disclosures were present (Table 3). Personal experience with alcohol use during pregnancy was mentioned by interviewees in all modalities, with slightly more disclosures in the in-person and online synchronous text-based modalities. No one in an in-person interview mentioned having to take medication for a mental health condition, though there was at least one such disclosure in each of the other modalities. The only mention of a previous abortion was in an online video IDI. Overall, sensitive disclosures were made by the greatest proportion (42%) of participants in the face-to-face IDIs.

**Table 3:** Frequency of disclosure of sensitive themes by modality

Interviews	In-person	Online Video	Online Chat	Email
	*n* = 12	*n* = 12	*n* = 12	*n* = 12
Alcohol use during pregnancy	5	2	4	3
Medication for mental health	0	2	1	1
Previous abortion	0	1	0	0
IDIs with at least one sensitive disclosure	42%	33%	33%	25%
	Fisher’s Exact Test,* p* = 0.97			

Focus groups	In-person	Online Video	Online Chat	Online Posts
	*n* = 6	*n* = 6	*n* = 6	*n* = 6
Alcohol use in pregnancy	5	5	5	4
Medication for mental health	3	3	4	3
Exposure to secondhand marijuana	1	0	0	0
Tobacco use	0	0	0	1
Previous abortion	0	1	1	0
FGs with at least one sensitive disclosure	100%	83%	83%	100%
	Fisher’s Exact Test,* p* = 1.00			

The focus group data showed similar trends across modalities. Alcohol use and medication for a mental health condition were present fairly consistently across all modalities. The differences appeared in those disclosures that occurred rarely—exposure to secondhand marijuana smoke, personal tobacco use, and having had an abortion previously—and were spread across the four modalities. At least one sensitive disclosure was made in 5 of 6 FGs (83%) for each modality. There were no statistically significant differences in overall sensitive disclosures by modality for either FGs or IDIs (Table 3).

### Dissenting opinions

This analysis included focus group data only and looked at one question on abortion to compare the frequency of dissenting opinions between text-based online modalities (non-visual connection) and in-person and online video modes of data collection (visually connected). In both online text-based modalities (chat and discussion board posts), at least one participant expressed a dissenting opinion on abortion in nearly all (5 of 6) groups (Table 4). In contrast, a dissenting opinion was raised in just half of the online video groups and in only one of the face-to-face groups. The non-visual, online text-based focus groups were 2.8 (95% CI: 1.2, 6.8) times more likely to contain a dissenting opinion (*p* = 0.01) than the “visual” in-person and online video focus groups.

**Table 4:** Frequency of dissenting opinions within focus groups and abstentions among participants

FGs	Visual		Non-Visual		Fisher’s Exact Test* (*p* value)
	In-person *n* = 6	Online Video *n* = 6	Online Chat *n* = 6	Online Posts *n* = 6	
# groups in which a dissenting opinion was expressed	1	3	5	5	0.04 (*p* = 0.01)

	Participant *n* = 27	Participant *n*= 33	Participant *n* = 33	Participant *n* = 38	
# individuals abstaining/not offering an opinion	0	6	4	8	0.31 (*p* = 0.25)

*Test of visual vs. non-visual modalities

We also assessed how many participants in each focus group modality abstained from this question. The percentage of abstaining participants was highest (21%) in the online posts, followed by the online video FGs (18%) and online chat FGs (12%). No one abstained from offering an opinion in the face-to-face groups. The differences in the rates of participants abstaining from the question on abortion were not statistically significant.

### Participant perceptions of data collection modalities

Participants rated their experience of the data collection event on four dimensions: (1) rapport with the interviewer/moderator, (2) creation of a safe space to talk, (3) comfort sharing honest experiences and opinions, and (4) convenience. No significant differences were identified in any of these domains among the IDI sample, while varying levels of statistically significant differences were observed in participant perceptions of the same characteristics of focus groups (Table 5). Across nearly all domains, women who participated in the online video FGs reported relatively lower levels of satisfaction.

**Table 5:** Participant perceptions of data collection modality

Interviews						
Question	Responses (%)	Face-to-Face	Online Video	Online Chat	Email	Total
		*n*= 11	*n*= 12	*n* = 11	*n*= 12	*n* = 46
Often in studies like this, researchers talk about rapport or the degree of connection and engagement in communicating. How would you describe the level of rapport between yourself and the interviewer?	A lot of rapport—I felt like we were highly engaged and connected	73	58	55	25	52
	Moderate amount of rapport—I felt like we were engaged and connected	27	42	45	75	48
	No rapport at all—I felt like we were not engaged and connected	0	0	0	0	0
Please respond to the following statement: I felt that the IDI provided a safe space to talk and express my feelings	Strongly agree	91	45	60	70	67
	Agree	9	45	40	20	29
	Neither agree or disagree	0	9	0	0	2
	Disagree	0	0	0	10	2
	Strongly disagree	0	0	0	0	0
How comfortable did you feel answering questions in the IDI?	Very comfortable	82	67	82	67	74
	Moderately comfortable	18	33	18	25	24
	Slightly comfortable	0	0	0	0	0
	Not at all comfortable	0	0	0	8	2
Do you feel that the online/face-to-face nature of this IDI …	Made you MORE willing to share opinions, experiences and information	64	25	45	50	46
	Had no effect on your willing to share opinions, experiences and information	36	75	55	50	54
	Made you LESS willing to share opinions, experiences and information	0	0	0	0	0
How convenient was taking part in this IDI for you?	Very convenient	36	50	55	75	54
	Moderately convenient	55	33	45	25	39
	Slightly convenient	9	8	0	0	4
	Not at all convenient	0	8	0	0	2

Focus Groups						
Question	Responses (%)	Face-to-Face	Online Video	Online Chat	Online Posts	Total
		*n* = 27	*n* = 31	*n* = 31	*n*= 29	*n* = 118
Often in studies like this, researchers talk about rapport or the degree of connection and engagement in communicating. How would you describe the level of rapport between yourself and the moderator? *	A lot of rapport—I felt like we were highly engaged and connected	67	39	45	72	55
	Moderate amount of rapport—I felt like we were engaged and connected	33	58	55	28	44
	No rapport at all—I felt like we were not engaged and connected	0	3	0	0	1
Please respond to the following statement: I felt that the FG provided a safe space to talk and express my feelings. **	Strongly agree	81	29	60	59	56
	Agree	19	58	40	41	40
	Neither agree or disagree	0	6	0	0	2
	Disagree	0	6	0	0	2
	Strongly disagree	0	0	0	0	0
How comfortable did you feel answering questions in the FG? ***	Very comfortable	85	23	81	83	67
	Moderately comfortable	15	71	19	14	31
	Slightly comfortable	0	6	0	3	3
	Not at all comfortable	0	0	0	0	0
Do you feel that the online/face-to-face nature of this FG… ***	Made you MORE willing to share opinions, experiences and information	54	35	84	76	62
	Had no effect on your willing to share opinions, experiences and information	46	42	16	17	30
	Made you LESS willing to share opinions, experiences and information	0	23	0	7	8
How convenient was taking part in this FG for you? ***	Very convenient	31	19	77	66	49
	Moderately convenient	46	58	16	28	37
	Slightly convenient	23	19	6	7	14
	Not at all convenient	0	3	0	0	1

*p < 0.05; **p < 0.01; ***p < 0.0001

#### Rapport

A substantial majority (73%) of women who participated in an in-person interview felt that rapport during the interview was high, with perceptions of a high level of rapport decreasing across the modalities from online video to online chat and finally email, where only 25% of women reported feeling a high level of rapport and engagement with the interviewer (Table 5). This differed from the focus group context, where both in-person and online message board participants reported feeling high levels of rapport, and a majority of both online video and online chat participants reported moderate rapport. No participants reported feeling “no rapport” in any of the individual interview modalities, while three women from online video focus groups reported feeling “no rapport”. Of the respondents who provided an open-ended comment, one explained that “It was hard to build rapport online for me,” while another, who noted moderate rapport in an online video focus group, stated that “There was a good bit of rapport, but I would say technical issues (like audio cutting in and out, video freezing) really disrupted it.”

#### Safe environment

Nearly all participants in individual interviews across modalities agreed or strongly agreed that the interview environment felt like a safe space to talk and express their feelings. The exception was one woman in an email interview who disagreed, stating, “[I] wasn't sure who or where these emails were going; I spoke my mind but was hesitant.” In the focus groups, the reported perceptions were similar; nearly all women agreed or strongly agreed that the focus group environment provided a safe space. Among the online video focus group participants, two women disagreed. One provided a reason, pointing to the group composition and topic, rather than the modality, per se, “One respondent was strongly against abortion and made me feel really uncomfortable about discussing my own feelings.”

#### Comfort answering questions and willingness to share

Nearly all participants in the IDIs felt at least moderately comfortable answering questions in all modalities; only one woman in the email IDI (same as above) felt not at all comfortable, citing uncomfortable questions. Focus group participants also reported high levels of comfort across modalities, with the exception of the online video focus groups, where the majority of respondents reported moderate comfort and two felt “only slightly comfortable”. Few comments were provided, but one woman’s response suggests the discomfort came from the nature of the questions as much as/rather than the modality, “It was still challenging to share opposing views, even knowing I would likely not see these women again, and even though everyone acted respectful during the video chat.”

Relatedly, women shared their perceptions on how the modality of data collection affected their willingness to share. Women who participated in online text-based IDIs were generally split between finding that the modality made them more willing or had no effect on their willingness to share their experiences. The majority of women in in-person IDIs felt the modality made them more willing to share, while the majority of online video IDI participants felt the medium had no effect on their willingness to share.

Within FGs, substantial majorities of participants in the online chat (84%) and online post (76%) text-based modalities reported that the mode of communication made them more willing to share. Women in in-person and online video focus groups were more evenly split between reporting more willingness to share and no effect on sharing. However, 23% of online video FG participants (and 7% of online post participants) thought the modality made them less willing to share, as summarized by one woman: “I did the online [video] focus group. If it had been just a telephone focus group, I would have been more open to sharing but seeing the other participants made me more nervous to be open.”

#### Convenience

The majority of women in all modalities reported that participating in the IDI was moderately or very convenient. One woman in the in-person sample and two in the online video sample felt the interview was less convenient; the only comment provided stated that as a full-time working mother of a toddler, there was no extra time for anything. The online text-based focus group participants generally reported those modalities as very or moderately convenient, while a greater proportion of in-person and online video focus group participants found the modalities moderately to slightly convenient.

Differences in responses by modality were statistically significant (*p* ≤ 0.05) for each of these domains in the FG sample, but none were significant within the IDI sample.

## Discussion

This study addressed questions about how changes in the modality of data collection might affect participants’ willingness to disclose sensitive personal experiences, dissenting opinions, and their overall comfort with the data collection process.

We found mixed evidence of an “online disinhibition effect” (Suler 2004) with regard to participants’ sharing of sensitive personal information across modalities. In terms of unsolicited sensitive personal disclosures, we found no statistically significant differences across modalities for either interviews or focus groups. It is difficult to determine the “usual” rate of sensitive disclosures in a qualitative data collection event, but we consider the range of themes and their presence across IDIs and FGs (and modalities) as an indication that these were salient issues. The relative frequency of mention of alcohol use during pregnancy may suggest that it is not a “sensitive” issue; however, in response to an earlier question that asked about “things a pregnant woman shouldn’t do to have a safe pregnancy”, drinking alcohol was mentioned by nearly everyone, so we considered personal disclosure of that behavior as going against social norms and therefore potentially sensitive. The more frequent disclosure of sensitive information in the focus group context aligns with earlier findings using a similar methodology (Guest et al. 2017b), though is subject to the same issues of different numbers of people represented in the unit of analysis (i.e., individual versus group of 7–8).

Evidence of an online disinhibition effect was observed in terms of focus group participants presenting dissenting opinions, which aligns with previous findings of more disagreement/willingness to dissent when visual cues are removed (Montoya-Weiss et al. 1998). Data on participant perceptions of the data collection modalities, which showed that the online video focus groups, in particular, were viewed as less comfortable and less of a safe space than the other methods, furthered this point. By contrast, greater proportions of women in the online text-based FG modalities, particularly the message board option, reported that the mode of data collection made them *more* willing to share, consistent with findings from Graffigna and Bosio (2006), who postulated there was also less social desirability bias evident in responses in their forum-based groups. Yet the data on abstentions—the number of participants in a group who chose to remain silent on a question—suggests that the anonymity of the online text-based modes of data collection provided participants more cover to “pass” on a question as well.

Findings on participant experiences of, and satisfaction with, different data collection modalities may reflect general use and familiarity with some modes of communication over others. The face-to-face IDIs and FGs both scored consistently high, with participants remarking on the conversational tone and ease of interpersonal exchange, even when discussing a sensitive topic. Given the similarity between face-to-face data collection and online video-based forms—spoken conversation, visual connection and ability to read non-verbal cues—it is perhaps surprising that online video FGs consistently received the lowest proportion of women reporting strong feelings of rapport, comfort, and safety. As participant feedback indicated, some of this relative discomfort was associated with technology issues that could interrupt the flow of conversation (freezing video, connection challenges), but women also seemed to “warm up” to each other less when connected via video versus in person. The pre-focus group small talk that happens in a conference room did not occur in online video contexts, and it may be that this helps participants to get a “read” on one another, identify similarities and connections, and set a more relaxed tone. Further, seeing oneself live on a computer screen among five to six others was cited as uncomfortable or distracting by a few women, in line with arguments put forth by Bailenson (2021) that “nonverbal overload” contributes to the phenomenon of Zoom fatigue. Like Archibald et al. (2019), we did not find a similar issue with video-based individual interviews.

Conversely, the relatively anonymous online discussion post modality scored high for the FGs in terms of rapport and comfort, perhaps reflecting women’s use of and familiarity with social media and posting platforms in other areas of their lives, and consistent with earlier conceptualizations of how people relate online (Montoya‐Weiss et al. 1998). We did not consider telephone (audio-only) data collection as part of this study given that telephone-based focus groups are uncommon, but the literature suggests the benefits of geographic reach and privacy, paired with speech, may make them a suitable option in some cases (Allen 2013; Koskan et al. 2014).

As with any research, our findings come with limitations. First, our study had relatively low statistical power to detect small to medium-sized differences between modalities, particularly when comparing focus groups. Our a priori definition of sensitive information may have differed from what individual women would consider sensitive, and the restriction of our analysis of dissenting opinions to one question provides a limited breadth of data. Also, our sample was relatively homogenous, limiting the generalizability of our findings. However, given our wider methodological objectives (Namey et al. 2020) and stratified participant assignment to data collection modality, sample homogeneity is also a strength in that it reduced a potential confounder in our comparative analysis. Relatedly, we recognize that the nature of the study and the experimental design necessitated eligibility criteria related to computer access and typing skills that may also introduce bias into the study vis-a-vis generalizability—that women who have home computers and can type are not representative of the general population of women who could benefit from remote data collection. Interpretations should be made with these limitations in mind.

## Conclusion

The Covid-19 pandemic motivated researchers across disciplines to use online qualitative data collection techniques, often for the first time. While many decisions were born of necessity, going forward, researchers will have the chance to be more intentional about the selection and use of online modes of data collection to supplement or replace in-person sessions. We have contributed empirical data points for consideration of the relative strengths and trade-offs of four modes of data collection for eliciting sensitive information and dissenting opinions, and in terms of participants’ experiences of the process. Though unsatisfying, there is no clear “right” choice of data collection modality, as it will always depend on research objectives, topic, and population. But knowing, for instance, that there may be an advantageous disinhibition effect for non-visual online data collection modalities can provide a starting point for assessing the options. Comparative research with more diverse populations—in terms of socio-economic status, race, computer/literacy, and mobility— and on topics of varying sensitivity would provide a broader base of empirical data to help guide study designs. And in the meantime, early engagement with individuals from the research population can provide a complement to existing literature by generating input on logistical, technological, or comfort/capacity concerns that might indicate a “better fit” option(s) for data collection.

## Data Availability

The qualitative data on which these analyses are based are not currently publicly available due to confidentiality concerns.

## Footnotes

1 *Face-to-Face and Online Synchronous Modality Procedures.* IDIs and FGs conducted face-to-face took place in a conference room at the study office and followed standard procedures for each of these two methods (Krueger and Casey 2009). Face-to-face focus groups also included an assistant who helped with greeting participants and refreshments. For all synchronous online activities (video and text-based chat), participants logged in to a private “chat room” at a designated date and time using an Internet-connected computer at home or in another convenient location. Online video events used web-connected video through this platform paired with audio over a conference call phone line so that all participants could see themselves, the interviewer and, in the case of FGs, all of the other participants. For synchronous text-based activities, the moderator and participant(s) typed questions and responses in real-time. Focus group sessions were conducted in “group mode”, where participants could see each other’s responses as they were entered. These three synchronous modes of data collection are referenced as in-person, online video, and online chat. *Online Asynchronous Modality Procedures.* Asynchronous, text-based IDIs were conducted over email, while FGs used an online discussion board platform. For IDIs, the moderator posed a series of three–five questions in an initial email and the participant responded according to her own schedule, within 24–48 h. The interviewer’s next email then contained follow-up questions and new questions. The same procedure was followed for asynchronous FGs: the moderator posted an initial set of questions on the discussion board and then added a few new ones each day over several days. Participants were instructed to sign in each day, respond to the questions, and read and comment on each other’s responses. The moderator reviewed message board activity several times a day and posed follow-up questions as appropriate, to which participants could again respond. Participants were prompted to complete unanswered questions before moving on to new ones. The asynchronous modality is referred to as email-based (IDIs) or online posts (FGs).

2 **Table:** Definitions of sensitive codes

Code name	Description. *Use this code when a participant discloses that she…*
Alcohol use during pregnancy.	… drank any amount of alcohol while pregnant.
Medication for mental health.	… takes or took medications for a mental health condition (e.g., depression, anxiety).
Exposure to secondhand marijuana.	… was exposed to secondhand marijuana smoke during pregnancy.
Previous abortion.	… personally has had an abortion in her lifetime.
Tobacco use.	… used tobacco products while pregnant.
